# Nanomedicine for COVID-19: the role of nanotechnology in the treatment and diagnosis of COVID-19

**DOI:** 10.1007/s42247-021-00168-8

**Published:** 2021-02-13

**Authors:** Farzan Vahedifard, Krishnan Chakravarthy

**Affiliations:** 1 Altman Clinical and Translational Research Institute, University of California San Diego Health Center, San Diego, CA USA; 2 Division of Pain Medicine, Department of Anesthesiology, University of California San Diego Health Center, 9400 Campus Point Dr, La Jolla, San Diego, CA USA

**Keywords:** Nanomedicine, Nanoparticles, Nanosensors, Severe acute respiratory syndrome coronavirus, Coronavirus 2019, Pandemic

## Abstract

Severe acute respiratory syndrome coronavirus 2 (SARS-CoV-2) has caused the recent outbreak of coronavirus 2019 (COVID-19). Although nearly two decades have passed since the emergence of pandemics such as SARS-CoV and Middle East respiratory syndrome coronavirus (MERS-CoV), no effective drug against the CoV family has yet been approved, so there is a need to find newer therapeutic targets. Currently, simultaneous research across the globe is being performed to discover efficient vaccines or drugs, including both conventional therapies used to treat previous similar diseases and emerging therapies like nanomedicine. Nanomedicine has already proven its value through its application drug delivery and nanosensors in other diseases. Nanomedicine and its components can play an important role in various stages of prevention, diagnosis, treatment, vaccination, and research related to COVID-19. Nano-based antimicrobial technology can be integrated into personal equipment for the greater safety of healthcare workers and people. Various nanomaterials such as quantum dots can be used as biosensors to diagnose COVID-19. Nanotechnology offers benefits from the use of nanosystems, such as liposomes, polymeric and lipid nanoparticles, metallic nanoparticles, and micelles, for drug encapsulation, and facilitates the improvement of pharmacological drug properties. Antiviral functions for nanoparticles can target the binding, entry, replication, and budding of COVID-19. The toxicity-related inorganic nanoparticles are one of the limiting factors of its use that should be further investigated and modified. In this review, we are going to discuss nanomedicine options for COVID-19 management, similar applications for related viral diseases, and their gap of knowledge.

## Pathology of COVID-19 infections

### Introduction

Severe acute respiratory syndrome coronavirus 2 (SARS-CoV-2) has caused the recent outbreak of coronavirus 2019 (COVID-19) [1]. This pandemic is challenging nations and has pushed policy makers to resort to social distancing steps and regular lockdowns [2]. The family of human coronaviruses (hCoVs) has caused three recent outbreaks [3]. However, it seems the death toll from four other contemporary epidemics (swine flu: 12,469 [4], SARS: 774, Middle East respiratory syndrome: 858 [5], and Ebola: 7588 [6]) was not high enough to sound the alarm for a pervasive pandemic, such as the current coronavirus pandemic. Until December 20, 2020, this nanosized virus of COVID-19 has infected 76.5 million people with a death toll of 1,690,000 and counting.

Currently, simultaneous research across the globe is being performed to discover efficient vaccines or drugs, including both conventional therapies used to treat previous similar diseases (e.g., influenza) [7] and emerging therapies (like nanomedicine) [8]. This virus may be testing our creativity in the ability to develop treatments for a new virus.

**Fig. 1:**
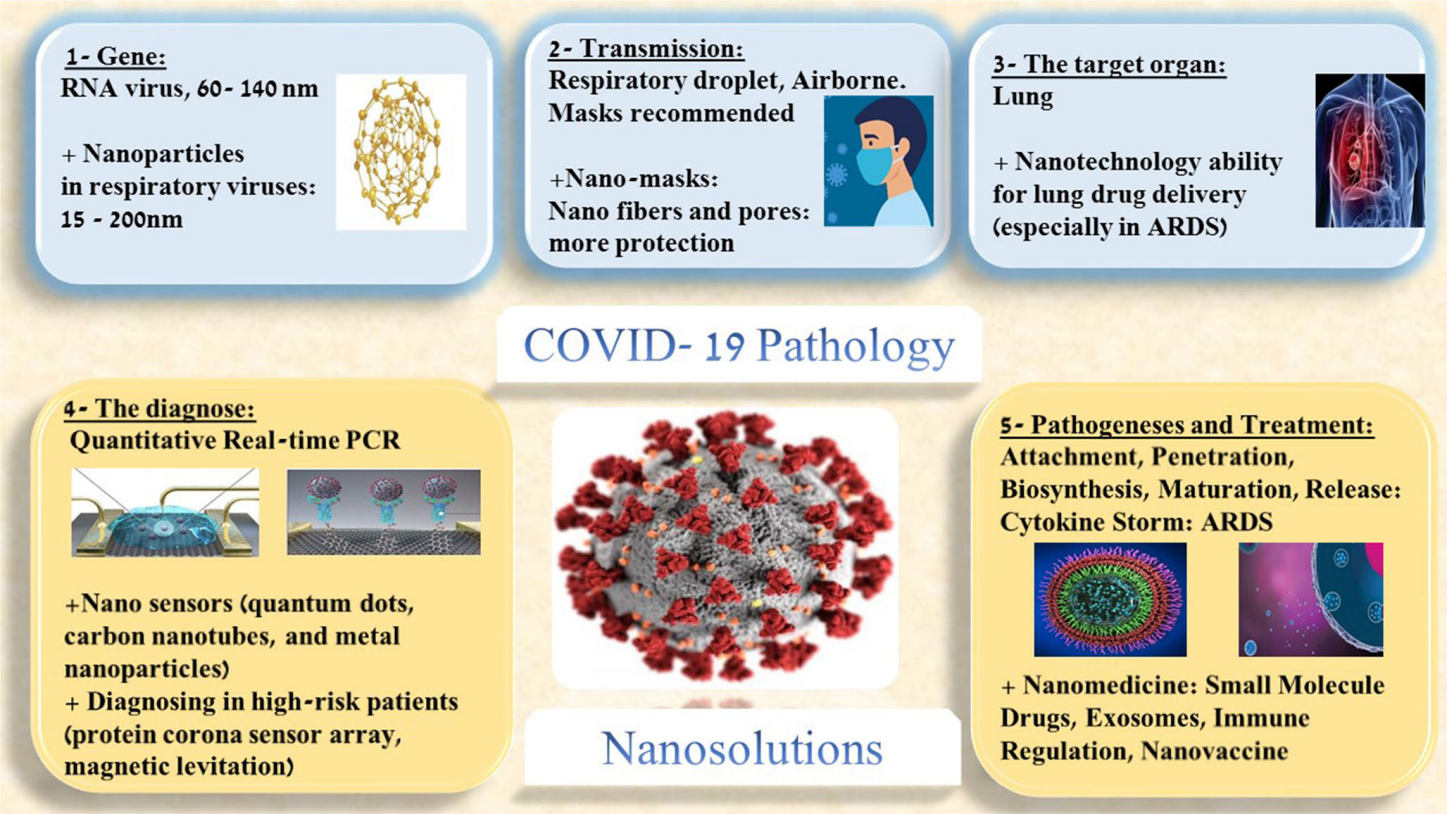
COVID-19 pathology and related nanosolutions

Nanomedicine has already proven its value through its application of drug delivery and nanosensors in other diseases [9–11]. In this review, we are going to discuss nanomedicine options for COVID-19 management. To do so, we will first examine the pathology of COVID-19 to set a foundation in order to expose opportunities and gaps within the pathophysiology of this virus, where nanomedicine could be of use (Fig. 1).

One of the advantages of this article is that it is written from the medical doctors’ point of view, who are directly in charge of a lot of patients with COVID-19 and also do work/research extensively in nanomedicine. The authors strongly tried to discuss the potential gaps and opportunities for management and diagnosis in COVID-19, through real clinical and basic science. In this way, we use cutting-edge findings, both in COVID-19 management, diagnosis, and nanomedicine.

### Gene

The COVID-19 genome is 80% similar to SARS (another virus that caused a pandemic in 2002), so it is named SARS-CoV-2. The COVID-19 is an enveloped, positive-sense, and single-stranded RNA virus [12]. It has a genome of 30 kb and a diameter of 60–140 nm [13].

Due to the virus’ small size, any mitigation method that can deal with it in a corresponding “nano” size would be more efficient. Tiny nanoparticles can move in the body without compromising other host functions, including the immune system [9, 14]. Confirming, the previous nanoparticles, which were used in respiratory viruses, have approximately similar sizes. These nanoitems include polymeric nanoparticles: 200–300 nm; self-assembling proteins and peptide-based nanoparticles: 15 nm; and inorganic nanoparticle: 100 nm [15, 16].

Nanoparticles have varied impacts and may affect other parts of the body. To reduce this risk, some researchers have focused on using iron oxide and magnetic fields to target specific organs (such as the lungs) for therapeutic purposes [17].

### Structure

The COVID-19 has various components that contribute to its pathogenesis: a spike (S) glycoprotein, a small envelope (E) protein, a matrix (M) protein, and a nucleocapsid (N) protein [18]. Specifically, the size of the spherical particles in this virus is between 70 and 90 nm [19]. Using electron microscopy, scientists can see extracellular and free particles [20]. The distinct envelope glycoprotein spikes, with 9–12 nm in size, exist on the external viral surface (Fig. 1). The 3D structure of the S protein of COVID-19 is also available at the RCSB PDB database (in both closed and open states) [21]. This structure is useful in designing therapeutics, vaccines, and neutralizing antibodies (such as passive immunization therapy, convalescent plasma therapy), probably through a B cell epitope [22].

Previously, nanomedicine has targeted several similar components in the treatment of similar respiratory viral diseases, such as the following:

1. In influenza: hemagglutinin [23], H1N1 antigen [24], HA split [25], antigen M2e [26]
2. In RSV: F and G glycoproteins, RSV phosphoprotein, Fsll [27]

### Transmission: preventive actions with nanomedicine

It is suspected that the initial transmission of COVID-19 was zoonotic, specifically through the seafood market [28]. Now, the human to human transfer of the virus plays an essential role in the spread [29]. Also, asymptomatic transfer between the carriers to healthy members of society are still an essential issue and a vital physiological and social challenge, as 1/3 of COVID cases can be silent [30]. However, the primary route of transmission of respiratory coronaviruses, including COVID-19, is the respiratory droplet [31]. The potency and rate of transmission in SARS-CoV-2 are remarkably higher in comparison to the other two species: SARS-CoV (R0: 1.7–1.9) and MERS-CoV (R0 < 1) [20, 32].

Therefore, wearing masks have been highly recommended for disease prevention during the COVID-19 pandemic [33]. The latest World Health Organization (WHO) recommendations have added airborne transmission to the possible COVID-19 transmission routes, especially during medical procedures. However, regular masks may not prevent COVID-19 airborne transmission completely. The quantity and quality of mask production have become a pressing challenge in this crisis, especially in the beginning stages. N95 masks can protect against most microorganisms; however, their quantity was low and in high demand even for medical personnel. Nanomasks, having extra layers of nanofibers and nanoscale pores, can prevent entering pathogens from the respiratory system. Thus, the efficiency of nanomasks in defending against virus transmission is much higher than ordinary masks [34].

Face masks, as well as lab or surgical aprons, have been nanoengineered to include new features, such as hydrophobic and antimicrobial activity, without compromising their structure or breathability of the material [35]. Hydrophobia of personal protective equipment (PPE) products can provide an important barrier against airborne droplets during coughing or sneezing. We can also use nanoscale 3D surfaces, material structuring, and hydrophobic nanoparticle coatings for this purpose [36].

Nanomaterials, such as nanofibers, can reduce breathing resistance and reduce pressure to provide comfort while at the same time defending against small particles (< 50 nm). This offers much greater protection than conventional surgical facial masks. Face masks N95 can only guard against particles 100–300 nm in size [37].

On the other hand, the mucous membrane is the entrance gate of the coronavirus into the human body. Consequently, the methods of prevention and delivery of drugs that work through the mucosa are critical. Nanotechnology has shown great potential regarding delivery across mucous membranes [38].

The safety of healthcare staff is also very critical in the case of a viral epidemic. The creation of infection-safe PPE for healthcare staff and the production of effective antiviral disinfectants is very important. Nano-based antimicrobial technology can be integrated into personal equipment for the greater safety of healthcare workers [39].

Contaminated objects in public areas, such as hospitals, parks, public transit, and schools, are a well-known widespread cause of infection [40, 41]. Some experiments have demonstrated the ability of nano-based surface coatings to eliminate infections [42, 43].

Many metallic nanoparticles are also considered to have a wide variety of activities against viruses and other microorganisms [44]. Rai et al. [45] reviewed the antibacterial, antifungal, and antiviral properties of metallic nanoparticles. Metallic nanoparticles, particularly silver (Ag) nanoparticles, may be used as a powerful and wide-spectrum antiviral agent with or without surface modification. Inhibiting viral entry, binding, or replication is the antiviral function of Ag NPs. In the cell-free and cell-associated virus, Ag NPs, for example, inhibit CD4-dependent virion binding, merging, and pathogenesis by interacting with the viral gp120. The Ag NPs in double-stranded RNA viruses can impede viral replication after contact with the viral genome [46].

### Entrance to body

The cycle of the COVID-19 within the host’s body involves five steps: (1) attachment, (2) penetration, (3) biosynthesis, (4) maturation, and (5) release [47]. In the final stage, the virus causes cells to undergo pyroptosis.

After the virus is attached to host receptors, a direct membrane fusion or endocytosis occurs between the coronavirus and the plasma membrane (penetration) [48]. Then, the virus’ RNA genome is released into the cytoplasm and enters the nucleus for replication. Once in the nucleus, the RNA genome begins transcription, translation, and protein synthesis (biosynthesis) [49]. The proteins are transported to the endoplasmic reticulum and the Golgi apparatus, maturated, and then released back into the body [50].

The S protein determines the diversity of coronaviruses [51, 52]. The angiotensin-converting enzyme 2 (ACE2) of the host cell receptor is the target for the receptor-binding domain (RBD). This S and ACE2 binding triggers endocytosis of the SARS-CoV-2 virion and exposes it to endosomal proteases [53]. The majority of ACE2 present inside cells (83%) are in alveolar epithelial type II cells, but they are also expressed on the cell surfaces in organs, such as the heart, endothelium, liver, kidney, testis, intestine, and possibly other vital organs [54].

Interestingly, various nanomaterials, including gold nanoparticles and carbon quantum dots (CQDs), are useful for interacting with viruses and preventing them from entering the cell [55]. They have a high surface-to-volume ratio and the ability to interact with a variety of functional groups [56].

### Target organs

The primary organ directly affected by COVID-19 is the lungs with typical symptoms of fever, dry cough, and dyspnea. However, the virus can enter the bloodstream through the epithelial cells of the respiratory tract, involving vascular systems and organs, for example, the brain, gastrointestinal tract, heart, kidney, and liver [57]. In the lung histology samples of COVID-19 patients, alveolar damage, desquamation of pneumocytes, hyaline membrane formation, and cellular fibromyxoid exudates were detected [58]. These characteristics limit the efficacy of the lung for gas exchange, which may lead to dyspnea, hypoxemia, vulnerability to infections, and susceptibility to acute respiratory distress syndrome (ARDS) [59].

The delivery of the drug to the lungs is compelling because of its significant systemic and local effects [60]. This point is essential in COVID-19, because of primary location involvement, duplication, and cause of death in the lungs. Nanotechnology has a great ability to directly deliver the drug to the lung, especially in ARDS. This capability has been confirmed in several papers [61].

### Pathogenesis

The pathogenesis of SARS-CoV-2 is similar to that of SARS, except that the inflammatory response is more severe in the new coronavirus. This more severe inflammatory response, along with the viral infection itself, is the leading cause of the disease’s severity [62].

Virus particles bind to the ACE2 and TMPRS2 receptors (a type II cellular transmembrane serine protease), which are expressed on the cells of multiple organs, notably pulmonary epithelial cells (on type I and II alveolar epithelial cells) [63]. The TMPRSS2 has a critical role in the activation of S protein and the fusion of the viral membrane into the host [64]. So, a possible treatment for COVID-19 is blocking the ACE2 receptor or TMPRSS2 in the host. The baricitinib (a Janus kinase inhibitor) [65], nafamostat, and camostat mesylate can inhibit TMPRSS2 [66].

In lung cells, COVID-19 binding to ACE2 receptors increases the expression of this gene, which increases permeability and leakage, leading to pulmonary edema, alveolar damage, and respiratory failure [67]. The virus, as it is released from infected cells, unloads damage-associated molecular patterns that signal nearby epithelial cells and alveolar macrophages to secrete pro-inflammatory factors, such as interleukin factors IL-4 or neutrophilic factors [68]. In most cases, these factors and recruited participants can elicit a relatively functional immune response that eliminates the virus before it spreads, resulting in a clearing of the virus within the lungs and patients' recovery [69].

However, in a dysfunctional immune response, multiple inflammatory mediators (IFN-α, IFN-γ, IL-1β, IL-6, IL-12, IL-18, IL-33, TNF-α, and TGFβ) and chemokines release, leading to a "cytokine storm" [70, 71]. Inside the lung, this storm leads to ECs dysfunction, inflammation, and vasodilation of the pulmonary capillary, even shock, and ARDS [72]. ARDS is a distressing condition associated with difficulty in breathing, pulmonary inflammation, and hypoxemic conditions [73]. In addition to making the patient more susceptible to secondary infections, ARDS can directly kill up to 70 percent of patients [74].

### Potential management

In general, COVID-19 medical approaches are divided into 4 actions: (1) vaccines; (2) targeting the CoV’s life cycle (preventing cell entry: camostat, mesylate, umifenovir; or inhibiting the cell cycle in the host cell: remdesivir, favipiravir); (3) targeting immune response: interferon beta, purified patient plasma; (4) prophylactic treatment: tenofovir-mefloquine; prevention of long-term injuries; and other treatments.

First, antibiotics, antimalarial, and antivirals are used to manage COVID-19 (azithromycin, lopinavir/ritonavir, chloroquine, hydroxychloroquine) [75]. However, their safety and efficacy have not been conclusively established in further studies [76].

Remdesivir (GS5734), an inhibitor of RNA polymerase that was previously used to treat viruses such as Ebola, was recently authorized for use in COVID-19 management [77]. It can decrease the duration of treatment of severe SARS-CoV-2 from 15 to 11 days [78].

In addition to the virus itself, the host patient’s inflammatory response plays an active role in the pathogenesis of the virus. If we can control this cytokine production and inflammatory response, we can reduce the disease’s severity [79]. Common methods used for similar diseases are also useful in treating ARDS caused by COVID-19: anti-inflammatory agents (corticosteroids, pharmacy nutrients, antioxidants, antiproteases), ventilator agents (neuromuscular blockers, β2 agonists, surfactants), diuretics, and anticoagulants [80]. The general treatments for ARDS and blocking IL-6, IL-1, and TNF in cytokine storms may be useful for COVID-19-induced ARDS.

Other medications have been used, such as α-interferon (e.g., 5 million units by aerosol inhalation twice per day) [81], sofosbuvir, and ribavirin [82]. Targeted immunotherapy is a good alternative for antiviral drugs with narrow treatment windows.

The viral components can be used as therapeutic targets. They included the underpinning subdomain, receptor-binding domain (RBD), and proteins, such as NSP7–NSP13 [83]. Using S protein, either full-length S protein or more intricate parts (S1 subunit, RBD domain, NTD, and FP), we can block the binding of the virus to the host receptor and viral genome uncoating.

Potential drugs for the treatment and management of COVID-19 are provided in Table 1.

**Table 1:** Some suggested and potential management of COVID-19

Antiviral	
Remdesivir (GS-5734™)	Nucleoside-based RNA polymerase inhibitor
Favipiravir [84]	Broad-spectrum RNA polymerase inhibitor
Umifenovir (Arbidol) [85]	Inhibits viral interaction and binding with host cells via ACE2
Chloroquine, hydroxychloroquine	Antimalarial drugs
TMPRSS2 inhibitor (camostat mesylate)	Protease inhibitor
Baricitinib	JAK inhibitor, prevent ACE2-mediated endocytosis
Inactivated convalescent plasma [86]	IV immunoglobulins
Immunomodulatory	
Steroid	Anti-inflammatory
Type I and type III interferons [87]	Antiviral, anti-inflammatory, and antifibrotic
MSCs [87]	MSCs
Tocilizumab, sarilumab [88]	A human monoclonal antibody, IL-6R antagonist
Anakinra [89]	A human monoclonal antibody, IL-1R antagonist
Other	
Heparin	Anticoagulant

Additionally, there are some emerging treatments for COVID-19, for example, (1) small-molecule drug-based therapies, (2) immune regulation therapy, and interferon utilizing (3) NK cell therapy, (4) exosomes, and (5) pluripotent stem cells (iPSCs) [90].

### Diagnosis

At present, quantitative real-time PCR (RT-qPCR) is used for confirming COVID-19 [91]. The real-time RT-PCR usually takes between 2 and 5 h. There are several places to extract samples, including nasopharyngeal and oropharyngeal swabs, sputum, lower respiratory tract secretions, stool, and blood [92]. Chest computed tomography (CT) is also the optional imaging method for COVID-19 [93].

New diagnostic methods have also been developed; for example, Abbott has developed a 5-min rapid test dependent on isothermal amplification of nucleic acid. This kit detects the RNA-dependent RNA polymerase (RdRP) of COVID-19 [94].

Also, serological testing, such as an IgM and IgG, is used to confirm COVID-19, which can display results after 10 to 30 days of infection, within minutes. However, we do not have an accurate interpretation of a person’s immune status based on the results of their antibody tests. These tests are also cross-reactive with other coronaviruses.

The timely diagnosis can lead to smarter, more efficient quarantine or distancing practices, lower socioeconomic burden, and prevent exacerbation of cases. Fortunately, nanotechnology can detect very low amounts of viral load. Employing nanoparticles for rapid and accurate detection of infectious agents in low-volume samples, at an affordable cost, helps early detection [95]. Various nanomaterials such as quantum dots (graphene quantum dots [96, 97]), carbon nanotubes, graphene oxide, silica, and metal nanoparticles are used as biosensors to diagnose pathogenic viruses like HTNV virus, RVFV virus, hepatitis A, B, E virus, herpes virus with Kaposi’s sarcoma, influenza virus A, human immunodeficiency virus (HIV), and human papillomavirus (H) [98].

### Prognosis

The severity of COVID-19 is varying from mild symptoms to ARDS, and fortunately, the most prevalent form is asymptomatic [99]. Men and Asian races are at a higher risk for COVID-19, probably because of higher levels of ACE expression [100]. The overall mortality rate of COVID-19 is reported at about 2.3%, with rates higher for the elderly than children [101]. Focusing on diagnosing high-risk patients with high mortality is a logical way to decrease mortality.

The novel nanotechnology methods, such as protein corona sensor array and magnetic levitation, can discriminate COVID-19-infected persons with a high risk of mortality in the early stages [102].

While complete quarantine of symptomatic patients is not possible in all societies, silent or asymptomatic COVID-19 carriers have more social interactions and, thus, a higher probability for disease dissemination. This is why the WHO and societies which were more successful in managing the disease are turning to countless and rapid COVID-19 tests.

### Vaccine

According to the WHO landscape of COVID-19 candidate vaccines (December 8, 2020) [103], 52 potential vaccines are in clinical evaluation, and 162 items are in preclinical evaluation. The platforms used for these vaccines included RNA, DNA, protein subunit, inactivated virus, nonreplicating viral vector, and virus-like particles. Some vaccines showed the promising effective result, published in December 2020. In December 2020, the Pfizer and BioNTech vaccine, as well as the Moderna Vaccine, received FDA panel votes approval for emergency use against COVID-19.

In pandemic conditions, mass production of the vaccine is required, usually not possible with conventional vaccine production. For this purpose, the novel methods of vaccine production can be helpful, such as next-generation sequencing, reverse vaccinology or genetic, immune informative, human challenge studies, and nanomedicine [104]. Interestingly, nanoparticle-based vaccines can induce a higher protective immunity response, rather than conventional antigen-based vaccines. For this purpose, gold, Ag, silver sulfide, titanium oxide, zirconium, and graphene have been suggested [105].

## Nanomedicine solutions (nanotechnology)

### Introduction

At nanomedicine, we have several tools that can address traditional antiviral medical limitations and other diagnostic/therapeutic measures. Drug delivery, cancer nanovaccines, immune engineering, nanosensors, and platform technologies are among the tools of nanoresearchers [106]. Due to the shortcomings of existing antiviral agents, which have a narrow spectrum and low bioavailability, nanomaterials can be a good option for antiviral treatment. Engineered nanomaterials (ENMs) have higher efficiency of drug delivery, fewer side effects, easily tunable physicochemical, and lower cost [107]. By changing the size, shape, and surface of ENMs, their interactions with virus particles or cell receptors can be adjusted to interfere with virus-cell interactions [108].

In this section, we will give an overview of the components of nanotechnology formulation related to COVID-19 and related viral diseases, as well as some of their important applications. Given that not many clinical trials with these nanocomponents are yet available, their applications in similar viral models could be very helpful. Finally, we will examine new and specific approaches to COVID-19 in nanomedicine.

### Different types of nanoparticles and their relevant applications

*Lipid-based nanoparticles* can be divided into liposomes, solid lipid nanoparticles (SLNs), nanoemulsions, and nanosuspension. They have several therapeutic applications with properties, such as large surface area, high drug-loading capacity, controlled release, and enhanced drug delivery [109].

SLNs have been used to deliver antiviral drugs, such as ritonavir, maraviroc, darunavir, efavirenz, zidovudine, and lopinavir. This application results in decreased first-pass metabolism and better tissue distribution [110].

Nanoemulsions (NEs), increasing aqueous solubility bioavailability and lymphatic uptake, can be conjugated with drugs, such as saquinavir or indinavir [111]. Lipid-based nanoformulations in the treatment of HIV [112] and VZV [113] also showed positive responses.

Lipid nanoparticles can also be investigated for gene silencing by RNA interference (RNAi) for antiviral therapy [114]. A type of small interfering RNA (siRNA) encapsulated in LNPs was tested to target the Ebola virus [115, 116]. It has been suggested that RNA interference may be effective in post-exposure therapy for the Ebola virus, and that it can be tested in other viruses.

*Polymer-based nanoformulations* (such as natural hydrophilic and synthetic hydrophobic) reduce the nonspecific interaction in the serum, so the pharmacokinetic parameters of the drug improve and side effects decrease by preventing early drug degradation. They were also effective for HIV, HSV, and hepatitis B.

Polymeric micelles have been successfully combined with efavirenz, darunavir, indinavir, acyclovir, and lamivudine. Other polymers, such as cellulose acetate butyrate or PLGA, have also been applied for efavirenz, nevirapine, and lamivudine.

Polymeric nanoparticles can target monocytes and macrophages in the brain and lymphatic system, as well as tackle viral diseases like HIV [117]. The combination of a polymer and a therapeutic agent can shrink or enlarge the therapeutic agent molecules [118]. The conjugation of interferon α2A and α2A with polyethylene glycol, with the exact mechanism, has been successful in the treatment of HCV [119].

The longer the chain of a polymer, the greater its viscosity (or resistance to flow like a liquid). They have a wider surface area, which makes them want to adhere to adjacent molecules. PEGylation has been widely researched and used by the methods used for nanoparticle protection to improve circulation times of nanoparticles. Polyethylene glycol (PEG) and PEG-like and other long-chain hydrophilic polymers have a flexible nature that helps minimize the adsorption of opsonin on nanoparticles and thereby allow them stealthy and unrecognized by our body’s removal mechanisms. Polymer chain length is proposed to be important in minimizing interactions between nanoparticles and opsonins [120].

*Dendrimers* are highly branched, well-defined, synthetic nanoarchitects [121]. They have excellent uptake by cells, longer circulation times, and targeted delivery [122]. Recently, glycodendropeptide has been mentioned as a promising viral treatment [123]. Also, dendrimers with inhibiting viral entry fusion have been successful in treating HIV and HSV2 [124]. Some of them, such as polyanionic carbosilane dendrimers, can act by binding to viral gp120 CD4 and CCR5/CXCR4 receptors at the host cell surface.

Polyamidoamine (PAMAM) dendrimers were effective in preventing influenza in mice [125]. Sulfated PPI dendrimers have prevented inhibiting the internalization of HIV-1 into epithelial cells [126]. Thiolated dendrimers loaded with acyclovir cause increased mucoadhesion [127].

Organic nanoparticulated dendrimers can also be used in the inhibition of the HSV-1/influenza virus. They can play a role in antibody-mediated response/CD8^+^ T cell activation, as well as gene expression through small RNA inactivation [128].

The mucoadhesive dendrimer and mucus-penetrating nanoparticles improve mucosal adhesion of the acyclovir [129]. The nanocrystals increase the bioavailability of nevirapine [130], and nanodispersion enhances the solubility of efavirenz [131].

*Nanocapsules* have an inner core, surrounded by the polymeric shell, which are used in targeted drug delivery and high drug loading. The nanocapsules, consisting of the poly core with entrapping azidothymidine triphosphate (AZT-TP), were used to deliver the drug directly to the cytoplasm [132].

*Nanospheres* are smaller in size (10–200 nm in diameter) with rapid drug clearance. Chitosan nanospheres loaded with acyclovir can treat herpes, which is more effective than acyclovir given alone [133].

*Peptides* from natural and microbial sources can have a wide range of antiviral properties. They have different sources, from frog skin, marine sources, plants, mammals, and bacteria. They were used for HIV, H1N1, DENV, and HCV [134, 135].

*Carbon nanoformulations* include carbon nanotubes (CNTs), graphene oxide nanoparticles, and fullerenes. CNTs are widely used in the delivery of chemotherapeutic drugs, which have a high pulmonary toxicity. The combination of protoporphyrin IX and multiwalled nanotubes have also been found to be effective in treating influenza. This compound inhibits the influenza virus through RNA strand breakage and protein oxidation. Also, functionalized CNTs with the transport of virus peptides are effective in producing the foot-and-mouth virus vaccine [136].

Graphene has a two-dimensional (2D) planar sheet and promising antiviral properties. Surface loading properties and superior mechanical strength make them suitable for transporting antiviral drugs [137]. Graphene can be effective in the Ebola virus by interaction with VP40 (viral matrix protein) of the virus [138]. Also, sulfonated magnetic nanoparticles combined with reduced graphene oxide (SMRGO) are effective in treating 28 viruses including HSV. Nanoparticles and gene-silencing technologies can be used to treat the respiratory syncytial virus.

Another symmetric carbon nanostructure is the fullerenes, which are fully composed of carbon atoms. They can fill the cavity of HIV proteases and prevent HIV proliferation [139].

Graphene oxide (GO) has antibacterial and antiviral properties, and inactivates the virus before entering the cell. Glycoprotein spikes in the virion envelope were destroyed after incubation with GO for 1 h. This property has been useful in DNA and RNA viruses. GO also establishes a negative charge, which interacts with a positive charge of the virus. GO negative inhibits herpes simplex virus type 1 infection, due to the presence of carbonyl and sulfonate surface groups. GO has also been used to identify and diagnose influenza A.

*Quantum dots* (*QDs*) (2–10 nm) are semiconductor nanocrystals, which have desirable optical and electronic properties. The QDs are used in sensing, imaging, and therapy of viral disease [140]. The conjugated quantum dot has been effective with anti-AIDS drugs and passes saquinavir through BBB [141].

QDs are normally composed of elements (such as Cd, Pb, and Hg) from groups II–VI, III–IV, and IV–VI in the periodic table, with sizes 2–10 nm. More recently, I-III-VI QDs (where I = Cu or Ag, III = Ga, or In, VI = S or Se) have been developed by researchers [142].

Different QDs can be used for diagnosis, such as CdSe (for CA-125 serum biomarker) [143], ZnO (ovarian cancer [144], and human T lymphoblastic cells by ZnO nanorods [145].

Nanoparticles in the form of metal (Ag and gold) and metal oxides (SiO_2_, TiO_2_, and CeO_2_) have broad antiviral properties against influenza, HSV, HIV-1, dengue virus type-2, HBV, and vesicular stomatitis virus [146].

Gold nanoparticles (AuNPs) have applications in biological imaging, electronics, and materials science [147]. AuNP can enter the cells where HIV is replicating, such as lymphocytes and macrophages. It also inhibits HIV replication in peripheral blood mononuclear cells.

AuNP can lead to viral deformation by mimicking a target binding receptor for the virus. These particles can be used to treat HSV, human papillomavirus, respiratory syncytial virus (RSV), dengue, and lentivirus [148]. Also, with the prevention of viral cell attachment and penetration, they have been useful in the treatment of HSV [149].

Small interfering RNAs (siRNAs), which are subject to degradation and rapid elimination, in combination with AuNPs, increase stability and decrease dengue virus replication and release [150].

Silver nanoparticles (AgNPs) can interfere with respiratory chain and electron transport chain enzymes are effective in fighting many viruses [151]. Due to the multiplicity of their targets, they are unlikely to be resisted. AgNPs are effective in treating AIDS. They can block gp120-CD4 interaction, as well as reduce the rate of reverse transcription in the post-entry stages of infection [152]. Also, they inhibit the attachment and entry of the HSV-1 virus [153]. These particles activate the inhibited agglutination of RBCs as well as the ROS-mediated signaling pathway in the fight against H1N1 [154]. In general, Ag and AuNPs are also effective in delivering therapeutic peptide [155].

Zinc oxide nanoparticles can boost T cell-mediated and Ab-mediated responses to HSV-2 and thus fight viral infection [156].

The superparamagnetic iron nanoparticles (γ-Fe_2_O_3_/Fe_2_O_3_/Fe_3_O_4_), in addition to the therapeutic applications, can inhibit viral multiplication even at the post-entry cellular level in viruses such as Zika virus, H_5_N_2_, and HCV [157].

Also, titanium nanoparticles (TiNPs) with properties, such as high solubility and photocatalytic activities, can have broad antiviral properties for bacteriophages and H_3_N_2_ viruses [158].

### Nano for diagnosis of COVID-19

Conventional detection methods used to detect COVID-19 based on nucleic acid detection, such as RT-PCR, sometimes have few limitations, such as low sensitivity, a large time needed to announce results, high false-negative, and lack of specificity due to similarity with other viral diseases [159]. Nanoparticles can solve some of these problems.

Nanotechnology can be used to rapidly detect COVID-19, which increases the efficiency of diagnostic tests [160]. Nanomedicine is effective in diagnosing organisms with high rates of resistance, which can be used as an alternative and prophylactic method for the rapid diagnosis of infectious diseases. Especially in new emerging infections, it helps in faster diagnosis and also lowers the cost of mass production in the fight against pandemics.

Metal and magnetic NPs, as well as quantum dots, are among the various nanoparticles that have been implemented for coronavirus detection. These materials are characterized by colorimetric, electrochemical, fluorescence, and optical detection techniques. These particles are synthesized in varying sizes (from 2 to 60 nm) have been used for several viruses in this family, for example, MERS, SARS, SARS-CoV-2, H5N1, and IBV.

Metal NPs, such as Au, Ag, and copper, have a special property called localized surface plasmon resonance (LSPR). Depending upon the morphology of these metallic nanoparticles, the SPR can be tuned from 300 to 1200 nm [161].

In the case of COVID-19, a combination of AuNPs with antisense oligonucleotides targeted the N gene, leading to aggregation of AuNPs induced by viral N gene [162].

AuNPs are the most common metal NPs used in coronavirus detection. AuNPs are the most common metal NPs used for coronavirus detection. For example, by using the colorimetric method, the difference between the electrostatic properties of single- and double-stranded DNA can be used to detect SARS-CoV [163]. Accordingly, Li designed a simple colorimetric hybridization assay to detect SARS-CoV FV based on the formation of dsDNA from viral ssRNA [26], which can lead to detection in 10 min without the need for additional tools.

Also based on disulfide formation reaction, change in the AuNP colloidal solution color in the presence of MERS-CoV can be found. These changes can be seen even at low concentrations, such as 1 pmol/μL.

AuNPs have also been used for electrochemical detection of coronaviruses. The spike protein S1 of MERS-CoV or Oc43 N of HCoV was immobilized to the surface of AuNPs. By adding the corresponding viral antibody, this connection leads to a reduction of square wave voltammetry (SWV) peak current. This method can detect MERS-CoV and HCoV in 20 min at low concentrations [30].

AgNPs were also used to detect MERS-CoV using paper-based analytical devices, which had a high sensitivity [164]. In this method, viral target cDNAs are evaluated for color change by the colorimetric assay method.

*Magnetic NPs* (*MNPs*): The most commonly used MNPs are iron oxide NPs, which have high magnetic efficiency [165]. These nanoparticles can be used to improve the Cdn detection of the SARS-CoV virus, similar to the method used in PCR-based assays for silica-coated superparamagnetic NPs (SMNPs) [166]. In this method, silica-coated SMNPs are bonded with oligonucleotide probes to identify irregular target cDNAs and lead to the formation of magnetic-conjugated dsDNA complexes.

A magnetoplasmonic NPs (MPNPs) can also be used with a combination of magnetoplasmonic-fluorescent biosensors based on zirconium QDs (ZrQDs) and Fe. In this method, the photoluminescence (PL) properties of the ZrQD nanohybrids were used to detect the virus [167].

Alternatively, the SARS-CoV-2 RNA may be detected and captured by a poly(amino ester) with a carboxyl-coated MNP group (pcMNP). The pcMNPs are magnetic, so the RNA complexes can be easily extracted by applying magnetic force [168].

The magnetoplasmonic nanoparticles and their combination with poly(amino ester) with carboxyl groups have also been used to improve RT-PCR in the diagnosis of SARS-CoV-2. This technique uses probe-functionalized MNPs to capture and enrich viral target RNA [169].

Biofunctionalized MNPs have been used extensively in the detection of pathogens because of their small size and excellent magnetic properties, which further limits biodetection time. The most commonly used MNPs are superparamagnetic NPs, which can detect more complex targets, such as viral particles in serum [170]. They are more portable than similar types and work at room temperature. We can use GMR biosensors for rapid detection system of the viral genome like +ssRNA and S (spike) protein [171]. They have high-sensitivity, less time consuming, low-costing device, compared to similar devices. The mechanism of action is the alternation of magnetization with changing electrical resistance from high to low and SARS-CoV-2 detection using GMR sensors [171].

Nanopore target sequencing (NTS) has also been used to detect COVID, and this method is based on the amplification of 11 virulence-related and specific gene fragments (orf1ab) of SARS-CoV-2 works [172]. Other parts included encoding S, ORF3a, E, M, ORF6, ORF7a, ORF8, ORF10, and N, which can also be targeted.

*Quantum dots* (*QD*), the nano-sized crystals, that have excellent nano-based sensing, are used to detect virulent pathogens. Different QDs with different metallic compositions (Pb, Cu, Ga, Zn, Hg) were used to detect HIV-1 and human T lymphotropic virus-1 [173]. They usually can be used, after their binding with NH2 receptor/biotin acceptor. Quantum dot aAPCs, for instance, were produced using commercially available nanocrystals coated with avidin. Antibiotin antibodies were functionalized, and the aAPCs were then manufactured using biotinylated peptide-MHC complexes. The quantum dot aAPCs can cause more than 15 times the expansion of inducing T cell proliferation when exposed to antigen-specific effector cells compared to noncognate controls [174].

The star-shaped chiroplasmonic AuNPs were combined with CdTe QDs to create a chiral optical biosensor. This method has been used to diagnose influenza A (H5N1), which is highly sensitive, and has been able to detect other viruses such as coronavirus [175]. In contrast to naturally occurring chiral materials, artificial chiral plasmonic nanomaterials have strong rotational potential to polarize light by designing intraparticle couplings with light-emitting nanomaterials at a nanoscale gap. In one research, this principle was used to build a chiroimmunosensor that employs the interaction of exciton-plasmon in chiral gold nanoparticle (CAu NP)-CdTe QD nanohybrids. The chiroplasmonic rough surface has more light-scattering properties and limited energy in space, and allows a tight coupling regime to be formed, increasing optical nonlinearities by correlations between light and matter with the QDs [176, 177].

Fluorescent QDs can also be used to detect coronaviruses. As mentioned, the binding of spike protein’s receptor to the host cell’s ACE2 receptor on the plasma membrane is one of the primary stages of COVID infection. Now, using QDs attached to the recombinant spike receptor, a versatile imaging probe can be created. Neutralizing antibodies and recombinant human ACE2 blocked quenching was a specific diagnostic reaction. This QD nanoparticle probe was also used to identify and validate inhibitors of the SARS-CoV-2 spike and ACE2 receptor binding. Therefore, these nanoparticles can be used for cell-based screening of inhibitors for coronavirus [178].

QD-conjugated RNA aptamer specific to the SARS-CoV N protein was highly sensitive to detect an immobilized viral protein [179].

Immunoassay strips are used to rapidly detect IgG antibodies against COVID [180], for example, a new rapid detection of IgM antibodies, based on a newly developed colloidal gold nanoparticle-based lateral flow assay [181] or dual-functional plasmonic photothermal biosensors for corona detection [182].

Other new reports on nanodiagnosis for COVID-19 have been published:

- Mertens introduced the Ag Respi-Strip diagnostic assay for this purpose [183].
- Another method by Zhao et al. works with pcMNPs. This method was able to work at 20 min and 10-copy sensitivity, which are promising results for the molecular diagnosis of COVID-19 [184].

Nanomedicine is used not only in PCR setting, but also in enzyme-linked immunosorbent assay (ELISA) and reverse transcription loop-mediated isothermal amplification (RT-LAMP) [185]:

- The combination of AuNPs conjugated with streptavidin has been used to diagnose MERS-CoV. Using a vertical flow visualization strip (RT-LAMPVF) assay, a biotin/fluorescein isothiocyanate (FITC)-labeled amplicon is fabricated. This combination can be seen with the naked eye in 35 min [28].
- Zhu et al. also introduced the NP-based biosensor system for COVID, which uses a “one-step and single-tube” reaction pathway, capable of delivering results in an hour. This system works with a single-step reverse RT-LAMP method combined with an NP-based biosensor (NBS) assay [169].

#### The advantages of nanomedicine in the diagnosis and mortality assessment of COVID-19

Several nanotechnology-based techniques are being developed to detect patients at high risk of mortality due to complications of COVID-19 infections, including blood clots. The important potential of nanomedicine in evaluating the risk of blood clotting and its severity in COVID patients has been shown. Also, its synergistic functions with advanced proteomics, focused on mass spectrometry approach for the detection of the essential protein of the occurrence and progression of COVID, were discussed [186].

Further, a new multidisciplinary, nano-based method has been proposed to detect COVID-19 infection in high-risk individuals in the early stages of the disease. This method, via techniques called protein corona sensor array and magnetic levitation, helps to prioritize patients properly and reduce the social and economic burden. These complementary diagnostic approaches also benefit from multiple analyzes.

Protein corona sensor array technology helps determine plasma protein/biomolecule patterns, which ultimately lead to fatal COVID-19 infection at very early stages.

Magnetic levitation (MagLev) is also a quick and easy diagnostic method, which helps to levitate nonbiological and biological species, incubated in the paramagnetic fluid [187]. For example, analyzing MagLev optical images can use machine learning techniques to identify “fingerprints” and to distinguish patients who are recovering from those who probably will expire [102].

### Vaccine

The vaccine is the most promising strategy to fight the coronavirus. Nanomedicine can participate in the design, delivery, and administration of COVID-19 vaccines because they have a multivalent antigen presentation and stabilization of antigens. They can also carry adjuvants to boost the immune response, and targeted delivery of antigens, such as an mRNA vaccine, which is delivered by a liposomal nanoparticle as one of the current vaccine candidates.

The conventional and subunit vaccines have limitations, such as reversion to pathogenic virulence or limited immunogenicity. But these problems can be solved by implementing nanoparticles and changing their properties, such as size, shape, and surface immunogenicity. However, some studies have raised the toxicity concerns of NPs, which require more attention to viral diseases [188].

NPs with properties and polymers, through faster mucous penetration that crosses the barrier and acting with little toxicity in the lungs, can be effective in treating respiratory diseases. Respiratory syncytial virus disease can be treated with virus-like NPs carrying RSV fusion proteins (F VLP). This method activates immune cell components such as natural killer cells, IFN-γ, and TNF-α.

Proteins such as inorganic NPs, self-assembly proteins, polymeric NPs, and peptide-based NPs are among the most common components of nanomedicine that can be used in COVID-19 vaccines. The peptide inhibitors and mutations of amino acids can play a role against SARS-CoV [189]. By binding to peptide ligand to a B cell epitope from the SARS-B HRC1 spike protein, we can neutralize the SARS-CoV infections via NP-based systems [190]. The peptide inhibitor isolated from ACE2 was able to block SARS-CoV-2. Its binding efficacy can be increased through multiple binding of nanocarrier-linked peptides [191].

More examples of using nanoparticles for the development of vaccines against viral diseases [192] are shown in Table 2.

**Table 2:** Some application of nanoparticles for vaccines of viral disease

Polymeric nanoparticles	Explanation	Virus target
PLGA	Poly(lactic-co-glycolic acid)	Swine influenza virus (H1N2)
γ-PGA	Poly-γ-glutamic acid	H1N1
Chitosan		H1N2, H1N1
Self-assembly proteins and peptide		
Nucleocapsid protein of RSV		Respiratory syncytial virus (RSV)
Ferritin		H1N1
Q11 peptide	Fibrilizing peptide	H1N1
Inorganic nanoparticles		
Gold		Influenza
Virus-like particles		RSV, H1N1, H3N2, H5N1
ISCOM	Quillaia saponin, cholesterol, phospholipid, and associated antigen	H1N1
DLPC liposomes	Dilauroylphosphatidylcholine	H1N1

In the field of vaccines, it is possible by activating the host immune system against the virus, using nanoagents. For this purpose, ENMs loaded with viral antigens can be used. These antigens include viral nucleic acid or inactivated proteins. For example, combining Au NPs with West Nile virus membrane proteins can release proinflammatory factors and antiviral immune response. Also, the combination of Au NPs with an anti-HCV DNA vaccine led to DNA vaccine uptake and improve antibody levels, and boosted immune response to HCV infection. Genetically engineered nanovaccines have also been used to successfully treat HIV-1, Ebola, and foot-and-mouth disease viruses.

The S protein could be a good target for the COVID-19 vaccine. For example, Moderna’s mRNA-1273 and BioNTech vaccines are now the types of mRNA vaccines that target the S protein of CoVs. The RNA and DNA vaccines have several advantages over older vaccines, with lower production costs and simple purification. However, the delivery of these vaccines to target cells is fraught with difficulties due to their stability and specificity, which nanomedicine-based delivery system can be effective here.

By combining RNA with nanocarriers, it can be effective in treating malignancies, infections, autoimmune disease, and neurological diseases by delivering small interfering RNA (siRNA), which also combines RNA-based vaccine mRNA-1273 with R drug substance, a nanocarrier system that is built. The nanocarriers that transport antigens reduce side effects by preventing the premature degradation of these molecules and also help to translate them with functional immunogens [193]. Nanocarriers can improve a molecule’s immunogenicity. Influenza antigen H1N1–conjugated chitosan NPs and *Yersinia pestis* F1-antigen–coated AuNPs, for example, developed higher levels of antibody and cytokine responses compared to mice administered with nonconjugated antigens [194]. This was shown to be due to vaccine antigen stabilization and improved immunogenicity due to conjugated antigens with NPs.

Vaccines using NP systems (NPb-Vs) can be delivered in two ways:

1. Antigen or RNA/DNA is encapsulated in a nanocarrier.
2. Attaching antigens on the nanocarrier surface, to be exposed to the environment.

Encapsulation with nanocarriers prevents proteolytic degradation of antigens and presents them directly to the APC [195]. After that, APCs take up the NPb. Then they translate the induced antigens to the cell surface, RNA, or DNA to the corresponding antigen. In this sense, RNA is preferable to DNA, as it can be translated directly into the cell cytoplasm, while DNA must reach the target cell nucleus.

On the other hand, NPb-V based on antigen encapsulation can exert a local depot effect, which prolongs the exposure time of antigen to the immune system.

Drug-conjugated nanoparticles have been effective in producing the influenza vaccine, with matrix protein 2 (M2e) and hemagglutinin (HA)/amine-functionalized gelatin surface coating.

Some of the suggested relevant nanovaccines are as follows:

- NanoFlu: a nanovaccine containing hemagglutinin protein nanoparticles has a high potential to stimulate the immune system against influenza (both common and altered strains). It is sufficiently safe in patients over 65 years of age [196].
- Novavax: a nanoparticle vaccine adjuvant with Matrix M, which uses full-length recombinant SARS-CoV-2 glycoprotein. It has the same platform for viruses such as CCHF, HPV, VZV, and EBV [197].
- Nuvec: using the technology of drug delivery, “Nuvec” can load COVID-19 plasmid and transfer it to the cell in a laboratory setting for vaccine formation. It is a silica nanoparticle with long spikes, and the entire structure is covered with a polyethylene glycol coating that causes a positive charge on the surface. Due to this unique topographic structure, the surface area has increased [198].

Another property of nanovaccines is that they can also be used in resource-poor or densely populated populations using their self-administration properties. Microneedle patches, single-dose slow-release implants, and film-based vaccines can be used for this purpose [199].

### Nanotreatment

The nanomedicine can work as antiviral agents, in varied ways (Table 3). We describe each of them separately.

**Table 3:** Some examples of nanosolutions for viral diseases, with their examples

Mechanism of action	Examples
Nanoparticles that block cell attachment and viral entry	• Titanium (Ti), silver (Ag), gold (Au), and zinc (Zn) • Cationic carbon dots (CDs) synthesized from curcumin (CCM-CDs) • Curcumin-modified AgNPs (cAgNPs) • Silicon NPs (SiNPs) • Mesoporous-SiO_2_ (mSiO_2_) • Selenium NPs • Graphene QDs can inhibit cell binding of HIV • GO and its derivatives
Nanoparticles that block viral replication and proliferation	• AgNPs and SeNPs • AuNPs • Ag2S • Benzoxamine monomer–derived CDs
Nanoparticles for viral inactivation and viricidal treatment	• • AuNPs • • Decoy virus receptor-functionalized nanodiscs • • Ferromagnetic Fe_3_O_4_ • AuNPs
Antiviral agent delivery	• Nanoencapsulation • Polymeric NPs • Gold nanoparticle-peptide triazole conjugates • PLGA-based NPs • Cationic liposomal NPs • The virus-mimicking nanoparticles • The polyamidoamines (PAMAMs)
Nanomedicine strategies to target the immune system	• Tocilizumab (an IL-6 antagonist) plus hyaluronate-gold NPs • Liposomal dexamethasone • LIF nano • Adenosine
Nanoparticles combining multiple approaches for treatment	• Combining carbon QDs (CQDs) • The multifunctional lipid-polymer hybrid nanoformulation • Virus-like particles (VLPs) and virosomes
Additional nanosolutions for COVID-19	• Regenerative nanomedicine • Mesenchymal stem cell secretome • Exosome

#### Nanoparticles that block cell attachment and viral entry

NPs can directly prevent receptor binding and cell entry of viruses, as well as viral internalization. For this purpose, nanoparticles with viral genome/core protein are merged and prevent viral replication/attachment release [200]. This method of inhibition is effective against dsRNA viruses and HSV-2 [201, 202].

The metal nanoparticles can block viral attachment to the cell surface and consequently prevent cell proliferation. Currently, nanoparticles, such as titanium, silver, gold, and zinc, have shown therapeutic results against HIV, influenza virus, herpes simplex virus, respiratory syncytial virus, transmissible gastroenteritis virus, monkeypox virus, and Zika virus [203]. The nanoparticles prevent them from interacting with the host cell by binding to their viral envelope or protein [204].

AuNPs can inhibit gp120 fusion to CD4.

The Ag NPs can bind to viral membrane proteins [205] and nuclei [206] interfere. AgNPs prevent primary infection with Kaposi’s sarcoma–associated herpesvirus by degrading the virion protein.

The virucidal properties of AgNPs have been shown in several other infections such as poliovirus type 1, coxsackievirus B3, and influenza A virus. Their mechanism of action can be through CD4-dependent cellular binding/pathogenesis or covalent linking with the sulfhydryl group at the virus level [207, 208].

AgNPs also have antiviral activity, and their major mechanism is through physical binding to the glycoprotein. Viruses that are bound to the nanoparticle are no longer able to penetrate the cell. Agglomeration of the AgNPs can be prevented by combining AgNPs with GO, as negatively charged GO may attract the viruses. The combination of GO-AgNPs has been used to treat a member of the coronavirus family called feline coronavirus (the most common deadly feline infection).

The combination of the GO-AgNPs also enhances the production of interferon-α (IFN-α) and IFN-stimulating genes (ISGs), which directly inhibit cell proliferation [8].

In a model similar to coronavirus, porcine epidemic diarrhea virus (PEDV) was investigated, in which cationic carbon dots (CDs) synthesized from curcumin (CCM-CDs) prevented the virus from entering the cell [209]. The mechanism of action is probably due to electrostatic interactions between the cationic CDs and the negatively charged PEDV, which causes virus aggregation. CDs also reduce apoptosis by suppressing the accumulation of reactive oxygen species.

Curcumin-modified AgNPs (cAgNPs) can also inhibit cell entry of respiratory viruses by having large and effective cell levels [210].

AuNPs can interfere with the cell entry mechanism. In the study of Huang et al., a peptide was identified, which could mimic the conformation of heptad repeat 2 (HR2) in MERS virus S2 protein. By doing so, HR1 interferes, preventing the cell fusion process. By immobilizing this peptide on the surface of gold nanorods, its inhibitory activity increased tenfold could completely block cell fusion, with excellent biocompatibility [211].

Silicon NPs (SiNPs) can also be scavengers of free virus particles and a potential treatment for COVID-19 [212].

Mesoporous-SiO_2_ (mSiO_2_) NPs can also inhibit the virus’ attachment to host cell receptors [213].

Iron oxide nanoparticles can interact with the S1-RBD protein receptor domain, which is used by SARS-CoV-2 in the infection of host cells and can be an alternative to COVID-19 therapy [214].

Selenium NPs have antiviral effects at high concentrations. By combining it with the antiviral drug Arbidol (ARB), cell entry of the influenza virus was effectively inhibited [215]. Cationic chitosan NPs, by targeting dendritic cells (DCs), can also block viral entry and be effective in vaccines [216].

Graphene QDs can inhibit cell binding of HIV [217], and AgNPs coupled with graphene oxide (GO) sheets can block cell entry of feline coronavirus (FCoV) and enveloped viruses [218].

GO and its derivatives also prevent viral infections by competing with cell receptors or with conformational damage of the viral protein.

#### Nanoparticles that block viral replication and proliferation

By reducing the replicating of the virus in the body, the body’s immune system can respond properly. Nanoparticles are effective in reducing the infectivity of the coronavirus family.

The influenza A virus, due to the numerous mutations and pandemics, is a good model for the study of COVID-19, which has been treated with several NP-based treatments. The Au NPs (PoGNPs), targeting the HA protein, inhibit viral infectivity and increased cell life from 33 to 96%. This inhibition was reconfirmed on H1N1, H3N2, and H9N2 viruses [219].

AuNPs functionalized with sialic acid–terminated glycerol dendrons can also inhibit viral HA protein. Combinations of drugs, such as AgNPs and SeNPs plus oseltamivir [62], AgNPs and SeNPs plus zanamivir [63], and SeNPs plus ribavirin, were investigated for inhibitory effects on H1N1.

Another model virus is the porcine reproductive and respiratory syndrome virus (PRRSV), which was well controlled by AgNP-modified GO [220].

Zika virus, which is similar to coronavirus and has led to a pandemic, can be treated with benzoxamine monomer–derived CDs. This compound has also been shown to inhibit encephalitis and dengue viruses, and other nonenveloped viruses [221].

In the pathogenesis and proliferation of the coronavirus family, the outer envelope with the surface proteins plays an important role. NPs can be effective in treating viral outer envelope by targeting them. Particles, such as AgNPs [222], AuNPs [223], porous SiNPs [65], and silica NPs [213], were used for this purpose. Enveloped viruses, such as VSV, HSV, and PRV, are well controlled with these particles. Therefore, the development of nanoparticle therapies to target virus envelopes may be very effective [153, 224].

As a model of COVID disease, AgNP was used for transmissible gastroenteritis virus (TGEV) and to reduce the rate of cellular apoptosis due to viral infection [225].

Ag_2_S nanoclusters also suppress RNA synthesis and cell proliferation of PEDV, another model of coronavirus. This method may work by activating interferon (IFN)-stimulating genes (ISGs) and cytokine expression [226].

#### Nanoparticles for viral inactivation and viricidal treatment

Inactivation or destruction of the virus itself is another target for treatment.

For example, AuNPs coated with 3-mercapto-ethyl sulfonate (MES) and its combinations can achieve irreversible inhibition of viruses, such as HSV, VSV, RSV, and dengue virus to HPV [148].

In another study, decoy virus receptor–functionalized nanodiscs inactivate the H1N1 virus. This substance, by targeting the virion’s surface proteins, caused irreversible physical damage to the envelope [227]. Interestingly, functionalized nanodiscs can cause self-disrupt of the envelope in the virus.

Ferromagnetic Fe_3_O_4_ NPs have been used for lipid peroxidation in the viral envelope, to destroy the integrity of viral surface proteins, including HA, neuraminidase, and matrix protein [228]. Also, these materials (called IONzymes) can be placed on the facemask, providing good protection against H1N1, H5N1, and H7N9.

The inhibition of the measles virus by AuNPs [229], inhibition of hepatitis C virus by AuNP-based nanozymes [230], and inhibition of H1N1 virus by bromide-coated silica NPs [231] were also reported.

#### Antiviral agent delivery

*Nanoencapsulation* of the proposed drugs can lead to a safer treatment of COVID-19 or other viral diseases. These particles can prevent the transcription, translation, and replication of various viruses, by causing irreversible damage to them. Drugs can be encapsulated in the nanocarriers, to target infected cells.

The benefits of nanocarriers include the following: (1) enhancing the solubility of certain drugs, (2) controlled sustained release of drugs results in longer therapeutic response, and greater exposure to the drug system (3) increase drug internalization and (4) target specific cells [232].

Chloroquine, as a suggested treatment for COVID-19, can block the endocytosis of SARS-CoV-2 virus particles. We can encapsulate the molecules of chloroquine, inside polymeric NPs, such as polylactic acid (PLA) polymeric NPs [233].

Nanotechnology solutions can also be used for the delivery of antiviral peptides. For example, Au nanoparticle-peptide triazole conjugates have been effective against HIV-1 because they disrupt the interaction between host receptor proteins and envelope spike glycoprotein of the virus [234]. Also, silver and Au nanoparticles plus an antiviral peptide FluPep have been effective in treating the influenza A virus [235].

*Nanocarriers* can be used to transport antiviral drugs. Organic nanoparticles have been used to deliver antiviral drugs such as zidovudine and acyclovir. The nanoparticle has improved bioavailability and drug delivery of them, and reduces the toxicity [236]. Interestingly, the prolonged circulation time of nanocarrier-based drugs is also effective for prophylactic treatment, especially for healthcare workers or those at high risk for CoV [237].

Acyclovir, which is used to treat HSV, has a shorter half-life (less than 3 h) and low bioavailability in oral administration [238]. Encapsulation of acyclovir into PLGA-based NPs can create a 2.6-fold improvement in drug bioavailability.

Using cationic liposomal NPs, the suramin can be boosted (used against HBV, HIV, ZIKV). The high negative charge suramin causes low membrane permeability and reduced cell entry, in which liposomes cause better inhibition of this drug [239].

We can also attach directly or conjugate antigens on the surface of nanocarriers [15], in which NPb-V mimics the virus itself. In this context, a specific immune response can be created by presenting S protein-specific antigens on the nanocarrier. The use of purification of immunoglobulins from patient plasma with this method against CoVs has been promising.

*The virus-mimicking nanoparticles*, such as self-assembled viral proteins, virus-like particles, or liposomes, can be used for the delivery system. Organic nanoparticles/nanocarriers are from the therapeutic compounds of large size molecule (> 10–1000 nm) delivery. These materials have been used for viruses such as RSV, HIV-1, and the Ebola virus. They also inhibit the SARS coronavirus (SARS-CoV), HSV-1, and human immunodeficiency virus (HIV of T cells) [240].

It is also possible to characterize the surfaces of nanocarriers for binding to CoV-related receptors.

The polyamidoamines (PAMAMs), a type of cationic NPs, via binding to the ACE2 receptor, can prevent the cleavage of angiotensin and ARDS [241]. Therefore, direct targeting of ACE2 or DPP4 can be a promising strategy in the treatment of COVID-19. We can target these receptors in the endocytosis pathway with a nanostrategy. Endocytosis is the main internalization mechanism for nanoparticles, and these drug delivery systems should be optimally sized for cellular uptake machinery (such as clathrin- and caveolae-dependent vesicles) [242].

The route of nanoparticle administration is also important; for example, in the systemic route, extravasation of the nanoparticle and its exit from the surrounding epithelial layer is a limiting factor. But the pulmonary administration via inhalation can be a good route to drug delivery to infected lung epithelial cells [243].

An intranasal delivery system, based on nanoparticles, is an efficient and secure mechanism for distributing a range of therapeutic components, such as vaccines, medications, siRNAs, peptides, and antibodies. Intranasal delivery is noninvasive, practical, easy, and cost-efficient and has been tested for vaccination of respiratory viruses such as influenza and CoVs [15].

In a new approach, we can use nanocarriers for clustered regularly interspaced short palindromic repeats (CRISPR) which is a high-precision and possible genome editing tool to be used in gene therapy. Tanaka et al. showed a viewpoint for the treatment of COVID-19 from the CRISPR/Cas9 system edition using protein modeling methods. The system uses a particular RNA guidance and a specific single-stranded oligodeoxynucleotide-mediated point mutation of the human ACE2 gene, involved in SARS-CoV-2 S protein binding [244].

#### Nanomedicine strategies to target the immune system

Various studies have shown that combination therapy of anti-inflammatory therapeutics and nanocarriers can increase their stability and resistance to degradation, improves targeted delivery, and drug exposure. For example, tocilizumab (an IL-6 antagonist) plus hyaluronate-Au NPs successfully treated RA [245], and dexamethasone acetate plus SLN acted successfully in delivering the drug to the lungs [246]. Also, the liposomal dexamethasone can act more effectively on multiple myeloma (https://clinicaltrials.gov/ct2/show/NCT03033316). The antifibrotic properties of dexamethasone can also be enhanced by nanoformulation.

In COVID-19, the nanoformulation can be used to target alveolar macrophages or phagocytes at the inflammation sites. Macrophages also have a proven role in the inflammatory response in various diseases, so can be therapeutically targeted in the inflammatory response caused by COVID-19.

Using a type of mesenchymal stem cell called LIF nano (leukemia inhibitory factor), which opposes the cytokine storm of viral pneumonia, the patient’s biological resistance to COVID-19 was improved [247].

As a new nanosolution, adenosine is used to treat cytokine storms. Adenosine is a powerful and natural anti-inflammatory substance produced in the body, but its direct injection can lead to adverse and severe side effects. By using drug-induced nanoparticles, adenosine is incorporated into a type of natural adipose tissue called “scalene,” which is packaged via a type of vitamin E called “alpha-tocopherol.” This method has been successfully tested on mice and could be used as a new tool to combat the uncontrollable inflammation caused by the cytokine storm in the COVID-19 [248].

#### Nanoparticles combining multiple approaches for treatment

Combined methods can create a synergetic effect and reduce the rate of virus infection. For example, combining carbon QDs (CQDs) of different sizes, with various functional groups, has been effective in the treatment of human coronavirus. Adding triazole, boronic acid, and amino groups to biocompatible QCDs, the inhibitory power for HCoV was enhanced [249].

In diseases such as COVID-19, where a single drug is not very effective, several drugs with different physicochemical properties can be placed in a delivery vehicle, such as lipid-polymer hybrid nanoparticles. The same thing is done with chemotherapy drugs [250]. This usage improves biocompatibility and controlled drug release.

With a single stable construct, different functions can be obtained, which is the mechanism of multifunctional lipid-polymer hybrid nanoformulation. Their applications can include detection, diagnosis, drug delivery, cell penetration, and destruction of the virus [251]. These compounds can deliver interferon-α, for the treatment of the hepatitis C virus, as well as elicit cellular and humoral immunity [252].

By changing the level of ligands of NPs, which mimic cell surface proteins, biomimetic lipid-polymer hybrid NPs can be achieved. These compounds have cell-specific targeting and attenuation of drug resistance [253], also can be used in the nanovaccines [254]. Virus-like particles (VLPs) and virosomes can be employed in this path. Using these compounds and glycoprotein fusion in the HA influenza, a more potent and broader response vaccine can be developed, than traditional influenza vaccines [255]. This self-assembling protein has been used to target the F protein of RSV [256].

#### Some additional nanosolutions for COVID-19

The morphology of the particle and metal surfaces, including spherical or nonspherical shapes (rods, prisms, triangles, tetrapods, dogbones, cubes, and shells) and physical properties, influences their efficacy. They also influence the frequency of plasmon band variance. The same effect has been found in the case of AuNPs and gold nanotubes (AuNRs) [257].

We can also use the regenerative nanomedicine for COVID-19, such as the mesenchymal stem cell secretome. In vitro evidence of cell-secreted extracellular material showed a variety of theoretical therapeutic effects on patients infected with COVID-19. MSCs can secrete IL-10, hepatocyte growth factor, and VEGF to relieve ARDS, regenerate, and repair lung damage against fibrosis [258]. MSCs can suppress the irregular recruitment of T lymphocytes and macrophages, resulting in their differentiation into T regulatory subsets and anti-inflammatory macrophages. These functional features of MSCs make them the optimal candidate among other available cellular therapies for the treatment of COVID-19 [259].

Further, exosome is a promising treatment regimen for COVID-19. Exosomes are small packets filled with cellular proteins and nucleic acid substances generated by stem cells [260]. They have shown regenerative capacity, immune-modulatory roles, and anti-inflammatory effects, close to those of their ancestors, mesenchymal stem cells [261, 262].

Four main areas where exosomes can be applied included:

1. Vaccine production and augmentation of innate and adaptive immune responses against SAR-CoV-2
2. Signaling pathways associated with hypoxia
3. Nanoscale drug delivery vehicle that reduces dosage requirement and toxicity while enhancing target specificity
4. Immunomodulation, which may reverse the cytokine storm—the main etiology of severe clinical symptoms of COVID-19 [263]

So the advantages of nanomedicine in treating COVID-19 generalized can be named as follows:

1. Potential to evade immune responses that would otherwise render treatment difficult
2. Homing capacity (the ability to deliver therapeutics to targets precisely—using complement opsonization or the conjugation of ligand to specific receptors on nanoparticle’s surface)
3. Benefits of using nanoscale particles, including but not limited to the enhanced permeability and retention (EPR) effect
4. Fewer toxic effects thanks to a lower level of accumulation of drugs in nontarget organs (nanocrystal)

The advantages of nanodisinfectants and health products over traditional products in the COVID-19 outbreak are also valuable, which include mass production speed, price, and the power of disinfection.

### Availability of nanomedicine in clinic

Currently, more than 50 nanomedicine drugs have been approved for various diseases [264]. Although the quantity and quality of nanomedicine production have increased, there are still two main issues regarding the availability of these drugs: first, the proper distribution of these drugs in pharmacies and hospitals, and second, the knowledge and awareness of the treating physicians to prescribe these drugs, as well as the pharmacists of how they work and their pharmacological details. This awareness can lead to better prescribing of these drugs, reduce treatment costs, and increase patient compliance.

Several studies were conducted in this area, especially in developing countries. For example, in 2018, the availability of nanopharmaceutical drugs in Palestinian hospitals, as well as pharmacist’s knowledge about these drugs, were examined. Only eight types of nanopharmaceutical drugs were available, and the frequency of availability of nanopharmaceutical drugs was low (between zero and 39%). Also in this study, pharmacist’s knowledge of pharmaceutical nanoformulations was lower than their knowledge of conventional drugs. Therefore, this study suggested that nanomedicine training courses in this regard be provided to medical and pharmacy students [265].

Further, there are multiple challenges to accelerate nanomedicine translation into the clinic. There are complex problems with the clinical translation of nanomedicine, such as a lack of business management education, especially at the academic level [266].

### Side effects of using nanomedicines

In general, toxicity-related inorganic nanoparticle is one of the limiting factors of their use that should be further investigated [267]. More tailorable biological properties can be created by making changes in size, shape, charge, or surface chemistry. To minimize the side effects of nanoagents, their physicochemical properties (e.g., surface modification) must be modified. Also, the dosage of the applied ENMs must be carefully adjusted. Also, the stability and bioavailability of antiviral nanoagents should be measured in different ways of drug application [107].

Although nanoformulation can reduce the side effects of drugs, their side effects should also be considered. Especially in nanomedicine, we use different manufactured nanoparticles made from various materials, and this can lead to more complications. The toxicity of these particles occurs at the molecular, cellular, and tissue levels. At the tissue level: inflammation—damage may occur; at the cellular level: reactive oxygen species—disruption of compartments; and at the molecular level: conformational change, loos of function, and aggregation are also possible [268].

`Toxicity for nanomedicines has been studied several times. For example, carbon-based nanoparticles have shown various laboratory and clinical toxicities [269, 270], and carbon nanotubes have been able to induce mesothelioma [271]. Interestingly, that toxicity was attributed more to its form, than the consequence of the actual materials [272].

The size of nanoparticles can also determine the biocompatibility of drugs. For example, Au nanoparticles were toxic at 1.4 nm, but not toxic at 15 nm [273]. Toxic effects on Ag nanoparticles have also been shown [274].

Another comparative study was performed between five pairs of drugs (one with nanomedicine formulation and one with conventional formulation). It was found that the toxicity of NMPs was not related to specific groups or structural types [275].

Most of the toxins found in NMPs were related to iron NP-related toxicity because iron can accumulate in the immune system [276, 277].

Various ways have been reported to reduce this toxicity. For example, the addition of hydroxyl groups to gadolinium fullerene particles can prevent the production of ROS [278, 279]. Also, the polymer coating of iron oxide nanoparticles can greatly improve cell viability [280].

The main step to reduce their side effects is selective targeting and preserving healthy organs. We can also use surface modification.

Of course, these studies do not determine the clinical guidelines sufficiently, because the number of drugs, as well as the number of people tested in studies, has not been sufficient. Therefore, further studies on the side effects of nanomedicine are recommended, especially in their interaction with the immune system.

### Regulatory of nanomedicine

The nanomedicine is followed by problems in the regulation of those materials. It is important to develop adequate knowledge about their efficiency, safety, and effectiveness, and to make consistent methods to help regulatory decision-making, for allowing a smooth transition into clinical applications [281].

Certain important geographical variations in the number of applications for marketing authorization for nanomedicine products have been verified in a recent study and raised the question as to whether the environment of certain regions is more appropriate for the marketing of nanomedical products than others.

There is no clear regulatory framework for nanotechnology-based products in different countries [282]. Variations reported in the approval process in these countries can arise from regionally different regulatory mechanisms and the classification of pharmaceuticals [283].

Initial papers with some guidelines regarding nanotechnology-based products have been published in countries such as the USA, Japan, and the European Union. They are not, however, legally binding documents and there is a lack of a specific regulation.

As there are still a small number of licensed nanomedicines in the majority of countries, which have heterogeneity of the product type, it is very difficult to obtain robust data sets allowing for general assumptions on the knowledge specifications relating to their consistency and safety [284].

The present lack of consistent terminology and categorization of nanomedicines complicates cooperation between agencies, which is another caveat for harmonized control of nanomedicines. For emerging products, developing a shared language is always a difficulty that should be resolved at an early level [285, 286].

Some countries are more advanced than others in the marketing of nanomedicines. These geographical disparities call for close coordination between the different regulatory bodies to exchange experiences and to train scientists, who in the future will be faced with more nanomedical applications. Further issues, such as the assessment of “nanosimilars,” borderline, and hybrid items, would require detailed regulatory understanding and are also on the international working groups [281].

In this regard, FDA announces: “FDA has issued several guidance documents on topics relating to the application of nanotechnology in FDA-regulated products. These guidance documents are being issued as part of the FDA’s ongoing implementation of recommendations from the FDA’s 2007 Nanotechnology Task Force Report. While guidance documents do not create or confer any rights for or on any person and do not operate to bind FDA or the public, they do represent FDA’s current thinking on a topic.” They include:

- Assessing the effects of significant manufacturing process changes, including emerging technologies, on the safety and regulatory status of food ingredients and food contact substances, including food ingredients that are color additives
- Considering whether an FDA-regulated product involves the application of nanotechnology
- Liposome drug products: chemistry, manufacturing, and controls; human pharmacokinetics and bioavailability; and labeling documentation
- Safety of nanomaterials in cosmetic products
- Use of nanomaterials in food for animals

### Role of nanoscience and nanotechnology roles for future infections

An important point to consider when dealing with pandemics is to work on prophylactic and novel strategies to deal with such infectious pandemics, which are just as important as the therapeutic approach. Therefore, different groups of scientists—especially in nanomedicine—are working on their production through bioinformatics tools.

### Future challenges in using nanomaterials

New therapeutics and vaccines can be given by nanotechnology to counter the current pandemic; nanomedicines, though, need to be proved secure first, as all modern treatments [287]. Also, they should be well tested before launching in the market.

The exact interaction of ENMs and therapeutic targets in the virus (mainly envelope, capsid, and nucleic acids) is not yet known. Also, the prediction and screening of interactions between viruses and ENMs require strong and high-throughput analytical platforms.

## Conclusion

Nanomedicine has already proven its value through its application drug delivery and nanosensors in SARS-like diseases. Nanomedicine and its components can play an important role in various stages of prevention, diagnosis, treatment, vaccination, and research related to COVID-19. Nano-based antimicrobial technology can be integrated into personal equipment for the greater safety of healthcare workers and people. Various nanomaterials, such as quantum dots, can be used as biosensors to diagnose COVID-19. Nanotechnology offers benefits with the use of nanosystems, such as liposomes, polymeric, lipid and metallic nanoparticles, and micelles for drug encapsulation, and it facilitates the overall improvement of pharmacological drug properties. Antiviral functions for nanoparticles can target the binding, entry, replication, and budding of COVID-19. The toxicity-related inorganic nanoparticles are one of the limiting factors of its use that should be further investigated and modified.

Biogenic NPs are eco-friendly, rapidly manufactured in large quantities, biocompatible, and of well-defined size and morphology. The close morphological and physicochemical similarity of SARS-CoV-2 with synthetic NPs also make NPs an effective intervention method. To perform specific functions, NPs may be extensively functionalized with different polymers and functional groups.

## Data Availability

The authors declare all data and materials support their published claims and comply with field standards.
